# Aberrant GLI1 Activation in DNA Damage Response, Carcinogenesis and Chemoresistance

**DOI:** 10.3390/cancers7040894

**Published:** 2015-11-27

**Authors:** Komaraiah Palle, Chinnadurai Mani, Kaushlendra Tripathi, Mohammad Athar

**Affiliations:** 1 Department of Oncologic Sciences, Mitchell Cancer Institute, University of South Alabama, 1660 Springhill Avenue, Mobile, AL 36604, USA; cmani@health.southalabama.edu (C.M.); ktripathi@health.southalabama.edu (K.T.); 2 Department of Dermatology, University of Alabama at Birmingham, 1530 3rd Avenue South, Birmingham, AL 35294, USA; mathar@uab.edu

**Keywords:** hedgehog signaling, GLI1, DNA damage, carcinogenesis, chemoresistance

## Abstract

The canonical hedgehog (HH) pathway is a multicomponent signaling cascade (HH, protein patched homolog 1 (PTCH1), smoothened (SMO)) that plays a pivotal role during embryonic development through activation of downstream effector molecules, namely glioma-associated oncogene homolog 1 (GLI1), GLI2 and GLI3. Activation of GLIs must be tightly regulated as they modulate target genes which control tissue patterning, stem cell maintenance, and differentiation during development. However, dysregulation or mutations in HH signaling leads to genomic instability (GI) and various cancers, for example, germline mutation in PTCH1 lead to Gorlin syndrome, a condition where patients develop numerous basal cell carcinomas and rarely rhabdomyosarcoma (RMS). Activating mutations in SMO have also been recognized in sporadic cases of medulloblastoma and SMO is overexpressed in many other cancers. Recently, studies in several human cancers have shown that GLI1 expression is independent from HH ligand and canonical intracellular signaling through PTCH and SMO. In fact, this aberrantly regulated GLI1 has been linked to several non-canonical oncogenic growth signals such as Kirsten rat sarcoma viral oncogene homolog (KRAS), avian myelocytomatosis virus oncogene cellular homolog (C-MYC), transforming growth factor β (TGFβ), wingless-type MMTV integration site family (WNT) and β-catenin. Recent studies from our lab and other independent studies demonstrate that aberrantly expressed GLI1 influences the integrity of several DNA damage response and repair signals, and if altered, these networks can contribute to GI and impact tumor response to chemo- and radiation therapies. Furthermore, the ineffectiveness of SMO inhibitors in clinical studies argues for the development of GLI1-specific inhibitors in order to develop effective therapeutic modalities to treat these tumors. In this review, we focus on summarizing current understanding of the molecular, biochemical and cellular basis for aberrant GLI1 expression and discuss GLI1-mediated HH signaling on DNA damage responses, carcinogenesis and chemoresistance.

## 1. Introduction

In 1980, developmental biologists Christiane Nüsslein-Volhard and Eric Wieschaus discovered the hedgehog (HH) signaling pathway in Drosophila (*Drosophila melanogaster*) [1]. Soon after this, three mammalian orthologs of HH were discovered, namely, Desert hedgehog (DHH), Sonic hedgehog (SHH), and Indian hedgehog (IHH). HH signaling plays a vital role in the differentiation of a variety of tissues in various organisms during development. Dysregulation of HH signaling leads to numerous differentiation defects like segment polarity, holoprosencephaly, microencephaly or cyclopia, absent nose or cleft palate [2,3]. Apart from its role in development and differentiation, HH signaling is also involved in regulating tissue homeostasis, cellular proliferation and stem cell maintenance in vertebrates [4]. Extensive analysis of HH functions has revealed contributions towards carcinogenesis, especially in basal cell carcinoma (BCC) and medulloblastoma [5,6]. Since glioma-associated oncogene homolog (GLI)s:GLI1, GLI2, and GLI3 are the final substrates that get activated by HH ligand, understanding their transcriptional targets and regulation has become an important focus of research. While the balanced physiologic activation of GLI1 regulates differentiation and development of various organs, mutations in HH signaling genes or oncogenic signals upregulate the expression of GLI1 in a non-homeostatic manner (aberrant GLI1) leading to the development of neoplasm. Somatic or germline mutations of GLI1 have been reported in several cancers [7]. Among the three GLIs, GLI1 was found to be the most significant target for cancer therapy, because its activation was found in many cancers by both HH signaling dependent and independent mechanisms [8]. In fact, the latter mechanism has been linked to several non-canonical oncogenic growth signals such as Kirsten rat sarcoma viral oncogene homolog (KRAS), avian myelocytomatosis virus oncogene cellular homolog (C-MYC), transforming growth factor (TGF)β, wingless-type MMTV integration site family (WNT) and β-catenin [9]. Interestingly, GLI1 inhibitors like GLI-antagonist 61 (GANT61) show superior anticancer activity compared to other upstream targets (such as HH or smoothened (SMO) inhibitors) of HH signaling [10]. Moreover, many agents that target upstream HH signaling are ineffective in suppression of GLI1 activity and their antitumor efficacies are limited to only certain tumors and largely ineffective in others [11,12]. Together these observations suggest an important role for aberrant GLI1 signaling in various stages of tumor development, from inducing mutagenic lesions, carcinogenesis, metastasis and resistance to cancer therapeutics. Recent research on the role of small molecule inhibitors of GLI1 and clinical trial studies on HH pathway antagonists has validated the members of this pathway as promising anticancer targets [10]. In this review we have discussed the role of aberrant GLI1 in DNA damage response (DDR), carcinogenesis and chemoresistance.

## 2. Molecular Mechanism of the HH Signaling Cascade

### 2.1. Canonical HH Signaling

**Figure 1:**
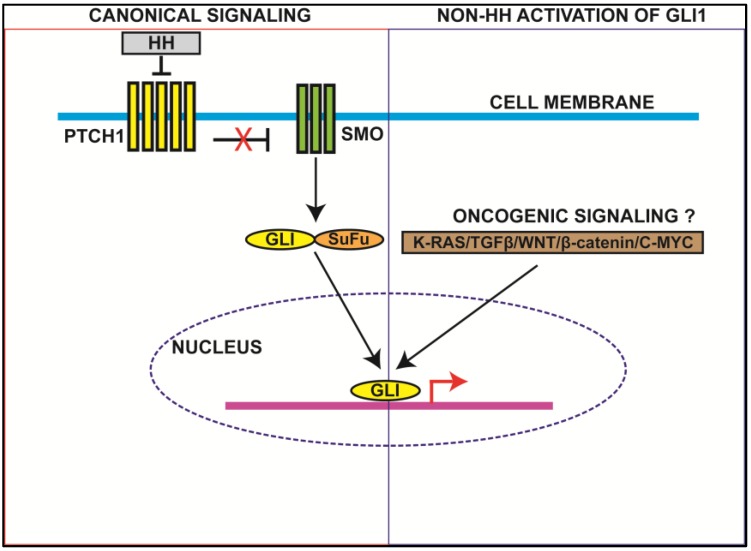
Canonical and non-canonical hedgehog signaling (HH) through glioma-associated oncogene homolog 1 (GLI1). Canonical HH signaling is activated by the binding of HH ligand to protein patched homolog 1(PTCH1), preventing its association with smoothened (SMO). This activates SMO leading to the dissociation of GLI and its translocation into the nucleus, where it serves as a transcription factor. In non-canonical activation of GLI1, various oncogenic signaling molecules, such as Kirsten rat sarcoma viral oncogene homolog (KRAS), avian myelocytomatosis virus oncogene cellular homolog (C-MYC), transforming growth factor (TGF)β, wingless-type MMTV integration site family (WNT), and β-catenin directly activate GLI1 in a Hedgehog-independent manner.

In the absence of HH ligand, protein patched homolog 1 (PTCH1), a twelve transmembrane protein receptor prevents the localization of SMO, a seven pass transmembrane G-protein coupled receptor-like protein, to the cell surface thereby inhibiting its activity (Figure 1). In canonical HH signaling, the HH ligand binds to PTCH1 receptor, triggering the phosphorylation and accumulation of SMO in the cilia [13]. Phosphorylated SMO then facilitates the dissociation of GLI proteins from kinesin-family protein, kinesin superfamily 7 (Kif7), and suppressor of fused (SUFU) [14]. Detachment of GLI proteins from Kif7 and SUFU is regulated by phosphorylation of GLIs by glycogen synthase kinase 3 (GSK3), casein kinase 1 (CK1), protein kinase A (PKA), Yak1-related kinase 1 (Dyrk1) and Slimb a F-box protein [15]. Upon phosphorylation, GLI1 protein translocates to the nucleus where it functions as a transcriptional activator and induces the expression of HH target genes such as *GLI1* (autoregulation)*, PTCH1, Cyclin D, MYC, B-lymphoma Moloney murine leukemia virus insertion region 1 (BMI1), B-cell lymphoma 2 (BCL2)* and *vascular endothelial growth factor (VEGF)* [16]. In addition to this, certain mutations in the HH signaling pathway members upstream of GLI1 induce its overexpression and alter the regulation of target genes that are involved in differentiation, DNA repair, and cell cycle checkpoint regulation.

### 2.2. Non-HH Activation of GLI1

In addition to the canonical HH-mediated activation of GLI1, several non-canonical signaling mechanisms have been implicated in regulation of GLI1 (Figure 1). For example, multifunctional cytokine TGFβ has been shown to elevate the expression of GLI1 independent of HH mediated signaling, but in a GLI2 dependent manner in different types of normal and cancer cells. These studies further elucidated that TGFβ-mediated expression of GLI2 depends on the functional SMA/MAD homology 3 (SMAD3) protein, suggesting TGFβ/SMAD3/GLI2 axis in regulation of GLI1 [17].

Further analysis of 5′-UTR (untranslated region) of GLI2 gene revealed TGFβ signaling mediated localization of SMAD3 and β-catenin, suggesting novel role for WNT/β-catenin signaling in regulation of GLI2 [18]. Another major HH independent regulator of GLI1 is KRAS. Activating mutations in KRAS have been linked to several cancers, including pancreatic, ovarian, lung and colon [19]. In most of the KRAS mutant tumors GLI1 was found to be overexpressed [20]. Functional studies have revealed the importance of GLI1 in KRAS-dependent pancreatic epithelial transformation and oncogenic activation [21].

Similarly, *C-MYC* was found to be another oncogene that activates GLI1 independently from HH ligand-mediated signaling [22]. C-MYC directly binds to the *GLI1* promoter and activates its transcription. Inhibition of C-MYC by small molecule inhibitors, downregulates GLI1 mRNA and induces cell death in Burkitt lymphoma cells. Similarly, aberrant expression of the transcription factor and oncogene EWS-FLI1, which is responsible for the Ewing sarcoma family of tumors, transcriptionally increases GLI1 expression [23]. A connection between GLI1 and p53 has been documented, e.g., loss of p53 results in aberrant GLI1 expression. On the other hand, studies have also shown co-regulation of GLI1 and p53 [24]. Furthermore, GLI1 regulates important oncogenes, including RAS, mitogen-activated protein kinase/Erk kinase (MEK), MYC, and AKT8 virus oncogene cellular homolog (AKT). The mechanisms of this regulation have not been thoroughly analyzed and further study could be important for understanding how GLI1 contributes to cancer development and may identify potential therapeutic targets.

### 2.3. Molecular Properties of GLI1

The GLI family consists of three transcriptional factors namely GLI1, GLI2 and GLI3. GLI1 has primarily activator function, whereas GLI2 serves as both activator and repressor, and GLI3 functions as a repressor. GLI proteins are present in both nucleus and cytoplasm, and upon activation, GLI1 and GLI2 enter the nucleus and activate the expression of HH target genes [25]. GLI1 belongs to the “GLI-Kruppel” family of zinc finger proteins, binds to the nine base pair conserved DNA consensus sequence 5′-GACCACCCA-3′ to regulate expression of its target genes [26]. Despite the fact that *GLI1* is a highly conserved gene in HH signaling, new mutations have been identified in this gene in different types of cancer [7,27]. Genetic analysis of pancreatic cancers showed mutations in GLI1 with clear functional relevance to neoplasia [7,27]. Missense and nonsense mutations of GLI1 were also documented in melanoma and squamous cell carcinoma [28,29,30]. Additionally, the fusion of *ACTB* (β-actin) with *GLI1* t(7;12), has been associated in pericytoma [31]. The strong *ACTB* promoter causes an overexpression of *GLI1* sequences and its downstream target genes. Apart from cancers, mutations in GLI1 were found in Hirschsprung disease, which is characterized as abnormal neural crest cells differentiation [32]. Germline GLI1 variations were also documented in the regulation of intestinal inflammatory pathways [33].

Recently, two splice variants of GLI1, GLI1ΔN (Deletion of 128 amino acids from the N-terminus) and tGLI1 (Deletion of 41 amino acids including the entire exon 3 and part of exon 4) have been identified [34,35]. Similar to wild-type GLI1, two splice variants of GLI1 (GLI1ΔN and tGLI1) follow the canonical activation by HH signaling. The (GLI1ΔN) variant acts on genes similarly to GLI1 in both normal and cancer cells. Interestingly, the tGLI1 variant is expressed only in the tumor tissues and has been characterized as a stronger promoter of epithelial-mesenchymal transition (EMT) phenotype, an important feature in carcinogenesis. Overall, aberrant expression of GLI1 and its isoforms in the setting of either canonical or non-canonical signaling regulates genes that are involved in the repair of DNA damage, cell cycle, carcinogenesis, and multidrug resistance (MDR).

## 3. Role of Aberrant GLI1 in DNA Damage and Repair

Human genomic DNA is under constant challenge by both endogenous and exogenous DNA damaging agents. It is estimated that each of the ~10^13^ cells within the human body incurs tens of thousands of DNA-damaging events per day. The cells have evolved with a wide array of DDR signaling and repair mechanisms to protect the integrity of the genome and faithfully transform the genetic information to the daughter cells. GLI1 influences the expression of several oncogenes that trigger cell division, regulates cell cycle check point and DNA repair mechanisms. Even though, many questions remain concerning how aberrant activation of GLI1 influences cell cycle checkpoints and DNA repair, there are few reports that clearly indicate potential mechanisms that GLI1 could control in order to protect tumor cells from oncogenic stress and chemotherapy. Likewise, regulation of GLI1 by various tumor suppressors and oncogenes in response to DNA damage, and convergence of these signaling molecules in deciding the cell fate is of great interest to understand the mechanisms that lead to genomic instability (GI), cell survival and death.

### 3.1. Regulation of GLI1 by DNA Damage

Studies have shown an important role for GLI1 in early carcinogenesis events in the activation of anti-apoptotic mechanisms in immortalized human keratinocytes with damaged DNA. Exposure of these cells to carcinogens induced more invasive growth in organotypic 3D cultures as well as in soft agar colony formation [36]. Further analysis revealed an increased expression of EMT markers such as snail family of zinc-finger transcription factor 1 (SNAILl) and vimentin and decreased E-cadherin in these cells. GLI1-induced expression of EMT markers in response to genotoxic lesions suggests a functional link between HH signaling and DDR, and transformation of precursor lesions to tumor development. These observations were further supported in genetic models of mice that are deficient in double strand break (DSB) repair genes. In this study, loss of either non-homologous end joining (NHEJ) gene DNA Ligase IV (Lig4), or genes involved in homologous recombination (HR) like X-ray cross complementation 2 (XRCC2), and breast cancer growth suppressor protein 2 (BRCA2), or (Lig4/XRCC2) in combination with p53 deficiency resulted in PTCH1 downregulation, GLI1 activation and rapid development of medulloblastoma [37]. This study not only confirms a function link between DDR, repair and HH signaling, but reveals that DNA damage induced expression of GLI1 was kept in check by p53 and loss of p53 results in GLI1 overexpression, a novel regulation of GLI1 by tumor suppressor protein p53.

**Figure 2:**
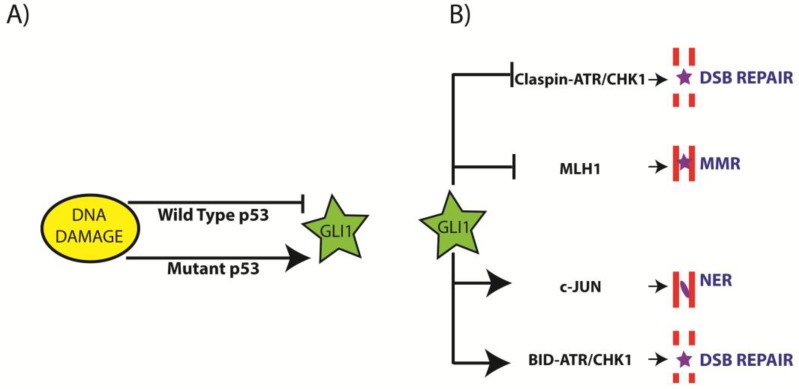
Role of glioma-associated oncogene homolog 1 (GLI1) in DNA damage response and repair. (**A**) Regulation of GLI1 by DNA damage. DNA damage increases expression of p53, which in turn activates the ubiquitin-mediated degradation of GLI1, suppressing cellular proliferation. In contrast, after DNA damage, cells lacking functional p53 are unable to suppress GLI1 in this manner and, therefore, are more proliferative; (**B**) Regulation of DNA repair by GLI1. GLI1 inhibits mismatch repair (MMR) and double strand break (DSB) repair through its regulation of MutL homolog 1 (MLH1) and ataxia telangiectasia-mutated protein kinase (ATR)/checkpoint kinase 1 (CHK1) signaling, respectively. At the same time, GLI1 activates nucleotide excision repair (NER) and DSB repair by regulating c-JUN and BH3 domain-only death agonist protein (BID)-ATR/CHK1 pathway, respectively. The magnitude of DNA damage and cell line individuality may decide the fate of GLI1 following DNA damage and its subsequent repair.

The above observations were further supported by mechanistic studies that demonstrated p53 mediated regulation of GLI1 by post-translational modification in response to genotoxic stress [38]. Exposure of human medulloblastoma cell lines and *Ptc^−/−^* mouse embryonic fibroblasts to DNA damaging agents, such as doxorubicin and cisplatin, induced concomitant expression of p53 and downregulation of GLI1 and its target genes. In these conditions, downregulation of GLI1 was found to be p53-dependent. The authors also observed a p53-dependent increase in an E3 ubiquitin ligase p300/CBP-associated factor (PCAF) in response to doxorubicin and cisplatin treatment. Furthermore, PCAF was found to ubiquitinate GLI1 in response to DNA damaging agents leading to its degradation, thereby preventing the growth and survival promoting activity of GLI1 overexpressing cancer cells. In response to damage, p53 induced cell cycle checkpoints prevents proliferation of damaged cells and provides sufficient time for repair. It is interesting to note that GLI1 is one of the primary targets of p53 in response to genotoxic stress, and degradation of GLI1 prevents the proliferation of cells. Moreover, absence of p53 failed to inhibit DNA damage induced GLI1 and its proliferation (Figure 2A). Overall, these results suggest that cancer cells have high genotoxic stress and the absence of p53 in these cancer cells may allow over expression of genotoxic stress induced GLI1, which makes it as an important therapeutic target in cancers with p53 mutation.

### 3.2. Inhibition of GLI1 Induces DNA Damage

Considering the importance of GLI1’s role in DNA repair, it is not surprising that inhibition of GLI1 induces DNA damage. In human colon carcinoma cells, inhibition of GLI1 induced extensive cell death while the inhibition of HH signaling at the level of SMO did not [39]. Inhibition of GLI1 by siRNAs or GANT61 showed increased DNA damage, activation of ataxia telangiectasia-mutated protein kinase (ATM)-checkpoint kinase 2 (CHK2) and cell cycle arrest at G1–S and in early S-phase in many tumor cells that aberrantly express GLI1. Moreover, GANT61 treatment induced downregulation of the genes involved in G1/S and G2/M progression such as E2F2, cyclin E2, Cdc25a, Cdk2 and cyclin A2, Cdc25c, cyclinB2, Cdc20, Cdc2 respectively [40]. Thus, inhibition of GLI1 blocks the replication-associated checkpoints and therefore, cancer cells with high proliferation rate and without proper cell cycle arrest/regulation will undergo cell death. GLI1 inhibition by continuous exposure to pharmacological inhibitor GANT61 for 48 h induced DNA damage and extensive cell death in colon cancer cells [41]. In these cells, the phosphorylated levels of ATM, H2AX, and mediator of DNA damage checkpoint protein 1 (MDC1) were increased; whereas, the level of phosphorylated nijmegen breakage syndrome 1 (NBS1) Ser343 was decreased. In contrast, cells that are exposed to GANT61 for only 24 h and then cultured in GANT61-free media were able to repair their DNA damage. Under these conditions, the levels of pATM, pMDC1 and pNBS1Ser343 were increased and the level of γH2AX was found to be decreased, suggesting inhibition of GLI1 by GANT61 attenuates DDR and repair. Interestingly, transient expression of pNBS1Ser343 alone in the cells that are exposed to GANT61 for 48 h caused increased DNA repair and cell survival. The authors concluded that decreased pNBS1Ser343 mediated DNA repair in 48 h GANT61 exposed cells lead to the cell death, whereas in cells exposed to only 24 h GANT61 treatment recovered from GLI1 inhibition mediated DNA damage due to the pNBS1 ser343 reexpression and its mediated DNA repair. These studies demonstrate an important role for aberrant GLI1 in regulation of the DDR and repair signaling in cancer cells, which could contribute to tumor cell survival from oncogenic stress and develop chemoresistance.

### 3.3. Regulation of NER by Aberrant GLI1

Cisplatin-induced intrastrand DNA crosslinks are primarily repaired by nucleotide excision repair (NER). The expression of genes involved in NER and base excision repair (BER) has been shown to be highly regulated by c-JUN [42]. Downregulation of GLI1 in cisplatin-treated ovarian cancer cells blocked the expression of c-JUN along with excision repair cross-complementing 1 (ERCC1), XRCC1 and xeroderma pigmentosum group D (XPD) (Figure 2B). Furthermore, GLI1 downregulated ovarian cancer cells treated with cisplatin showed a switch in the phosphorylation pattern of c-JUN from Ser63/73 to Thr91/93. Similarly, shRNA-mediated downregulation of GLI1 in ovarian cancer cells resulted in sensitizing these cells to cisplatin; reducing the IC50 value from 30 µM to 5 µM. To date, five different GLI1 isoforms have been described. The full length GLI1 isoform (GLI1FL) has a molecular weight of 160 kDa is the predominant variant. There is a truncated GLI1 (tGLI1) that is slightly smaller at 155 kDa and has only been found in tumors where it promotes an aggressive cancer phenotype. There are also two isoforms truncated at the N-terminus, GLI1ΔN and N’ΔGLI1, which are 140 kDa and 130 kDa, respectively and a C-terminal truncation variant called C’ΔGLI1 that is the smallest isoform at only 100 kDa. Molecular studies on the 5 identified isoforms (including two splice variants) of GLI1 revealed that expression of a 130-kDa isoform is involved in upregulation of c-JUN [43]. Mechanistically, it was found that only the 130-kDa isoform of GLI1 binds to the promoter region of c-JUN and regulates its expression. Further analysis revealed that this particular 130-kDa isoform of GLI1 is expressed only in the ovarian cancer cells and not in normal ovarian cells. Interestingly, the cisplatin-resistant ovarian cancer cells showed increased 130 kDa isoform expression compared to the other ovarian cancer cells.

### 3.4. Regulation of Mis-Match Repair (MMR) by Aberrant GLI1

In pancreatic cancer cells, GLI1 and GLI2 have been shown to control the expression of MutL homolog 1 (MLH1), a protein necessary for MMR and important for faithful DNA replication (Figure 2B) [44]. GLI1 transcriptionally enhances the expression of BHLHE41/DEC2/SHARP1, a basic helix-loop-helix type suppressor, which in turn suppresses the transcription of MLH1. Consistent with these studies, increased expression of GLI1 correlated with the decreased MLH1 in histological analysis of precancerous pancreatic intraepithelial lesions.

### 3.5. Regulation of DSB Repair by Aberrant GLI1

Impaired SHH signaling has been shown to induce spontaneous and ionizing radiation (IR)-induced genome instability and tumors development in mice [45]. When cells are exposed to DSB-inducing agents such as IR, several DDR signaling events including ataxia telangiectasia-mutated and Rad3 related protein kinase (ATR) and checkpoint kinases 1 (CHK1) mediated pathway gets activated. Although mutations in *PTCH1* in humans and *Ptc* heterozygous mice (*ptc*^+/−^) known to develop increased spontaneous medulloblastoma; however, the mechanisms that lead to tumor development were not known. It was demonstrated that elevated GLI1 in these mice abrogates ATR-CHK1-signaling, induces spontaneous and IR-induced genome instability and promotes tumor development. These *in vivo* studies were further confirmed by transient expression of GLI1 in human embryonic kidney (HEK) 293 cells. GLI1 expression abrogated IR-induced ATR-CHK1 signaling in these cells by disrupting DNA damage-induced binding of claspin to CHK1 and subsequent phosphorylation of CHK1 by ATR at Ser317 and Ser345 (Figure 2B).

Moreover, a mutant GLI1 lacking five zinc finger DNA-binding domains did not suppress IR-induced CHK1 phosphorylation, confirming GLI1 transcriptional activity mediated regulation of CHK1 phosphorylation. Thus, these studies imply a possible but important mechanism leading to carcinogenesis due to impaired HH signaling.

In contrast to above mechanism, our recent studies have revealed a tumor-specific role for aberrant GLI1 in regulation of S phase checkpoint [46]. Inhibition of GLI1 in several tumor cell lines originated from different tissues induced replication associated DNA damage as indicated by γH2AX and attenuated tumor cell growth. Further analysis of replication stress-induced by DNA topoisomerase 1 (TOP 1) poison CPT revealed aberrant GLI1 important for the activation of S-phase checkpoint mediated by CHK1 in tumor cells. We found that GLI1 transcriptionally regulates BH3 domain-only death agonist protein (BID), which localizes to the nucleus in response to fork-stalling lesions and facilitates the association of replication protein A (RPA) with the ATR- interacting protein (ATRIP)-ATR complex, and phosphorylation of CHK1. Furthermore, inhibition of GLI1 by either siRNAs or by pharmacological inhibitor GANT61 transcriptionally repressed the expression of BID, affected the association of RPA with the (ATRIP)-ATR complex, and compromised ATR-mediated phosphorylation/activation of CHK1.

Deficiency in S-phase checkpoint prevents DNA damage-induced cell cycle arrest and allows cells to progress through the cell cycle with damaged DNA, which may lead to mitotic catastrophe and cell death. Thus, Top1 and CHK1 inhibitors exhibit synergy in their cytotoxic effects. Similar to CHK1 inhibitors, pharmacological inhibition of aberrantly expressed GLI1 in tumor cells abrogated CPT-induced checkpoint responses, enhanced CPT-induced replication-mediated DNA damage and increased its cytotoxicity. Importantly, our data suggest the benefits of using the combination of GLI1 and Top1 inhibitors to treat tumors that over express GLI1. Moreover, DNA replication checkpoint mediated by CHK1 plays an important role in cell survival in response to oncogenic growth signaling by suppressing replication stress and resistance to chemotherapeutics by promoting timely repair of DNA lesions. Since most tumors are driven by oncogenic growth signals (such as KRAS, epidermal growth factor receptor (EGFR) *etc.*), our studies suggests role for aberrant GLI1 during carcinogenesis, as well as their response to chemotherapy.

### 3.6. Section Summary

DNA repair is frequently dysregulated or modified in cancer, making it important to analyze the impact of GLI1 on the differential regulation of these DNA repair mechanisms. GLI1 was found to repress the phosphorylation by CHK1 through two distinct and somewhat conflicting mechanisms. Firstly, overexpression of GLI1 blocks CHK1 activation by preventing the association of CHK1 with Claspin. Secondly, depletion of GLI1 causes a loss of BID which also leads to disruption of CHK1 activation. These seemingly incongruous findings could due to a variety of factors such as cell type; the pathway through which GLI1 is being regulated, canonical *versus* no-canonical activation; or other mutations present in DNA repair proteins. These findings emphasize the need for further study of the role of GLI1 in the DNA damage response. As disrupted DNA repair can directly lead to persistence of mutations that may promote carcinogenesis, studying GLI1 could lead to improved targeted therapies in repair-deficient tumors.

## 4. Role of Aberrant GLI1 in Carcinogenesis

As previously described, GLI1-mediated dysregulation of DNA repair can directly or indirectly lead to GI and such GI can promote carcinogenesis. Early studies of GLI1 in DNA repair and cancer focused on the canonical HH signaling pathway; however, recently a non-canonical pathway for activation of GLI1 by oncogenic proteins has been elucidated.

### 4.1. Aberrant GLI1 in Multiple Cancers

In sporadic BCC tumors, overexpression of GLI1 independent of upstream HH signaling molecules such as SMO or PTCH1 has been documented [6]. Furthermore, it was found that EGFR acts synergistically with GLI1 to induce oncogenic transformation in BCC [47]. These findings also reveal that in addition to the activation of JUN/AP1, EGFR also promotes nuclear translocation of GLI1.

**Figure 3:**
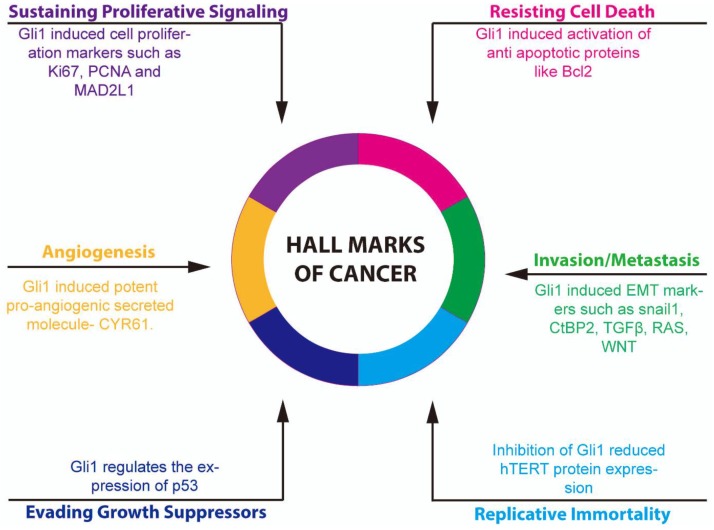
Role of glioma-associated oncogene homolog 1 (GLI1) in carcinogenesis. GLI1 regulates processes involved in all six of the traditional hallmarks of cancer. GLI1 protects against apoptosis by inducing anti-apoptotic proteins, such as B-cell lymphoma 2 (BCL2). GLI1 promotes cell invasion and metastasis through induction of epithelial-mesenchymal transition (EMT) markers such as SNAIL1, C-terminal binding protein 2 (ctBP2), transforming growth factor β (TGFβ), rat sarcoma viral oncogene homolog (RAS) and wingless-type MMTV integration site family (WNT). Replicative immortality can be achieved through GLI1-mediated regulation of human telomerase reverse transcriptase (hTERT) protein expression. GLI1 can aid in evasion of growth suppressors by regulating p53 and promotes proliferation by inducing expression of Ki67, proliferating cell nuclear antigen (PCNA) and mitotic spindle assembly checkpoint protein L1 (MAD2L1). Finally, GLI1 stimulates new blood vessel formation by enhancing expression of the potent pro-angiogenic protein cysteine-rich protein 61 (CYR61).

Human melanoma stem cells with increased aldehyde dehydrogenase 1 (ALDH1) activity showed greater expression of GLI1 [48]. Blocking GLI1 in these stem cells reduced tumor initiation and stem cell renewal. The overexpression of GLI1 in prostate cancer was found to correlate well with levels of stathmin1, a protein expressed in developing mouse prostate and prostate cancer, that functions as a regulator of mitotic spindle assembly [49]. Microarray analysis of gene expression in GLI1 overexpressing rat kidney epithelial cells (RK3E) showed altered levels of the cell cycle genes, cell adhesion genes, signal transduction genes and genes regulating apoptosis [50]. Furthermore, the zinc finger transcription factor GLI1 binds to the promoter and regulates expression of several genes including *cyclin D2*. Induced expression of GLI1 in HaCaT cells caused increased levels of cell cycle genes, and cell proliferation markers such as Ki67, proliferating cell nuclear antigen (PCNA) and mitotic spindle assembly checkpoint protein L1 (MAD2L1) (Figure 3) [51]. Additionally, increased GLI1 was found in several tumors such as gastric cancer [52], gastrointestinal stromal tumors [53], merkel cell carcinoma [54], pulmonary adenocarcinoma [55], bladder cancer [56], head and neck squamous cell carcinoma [57], melanoma [58,59] chronic cholecystitis, gallbladder carcinoma [60] and non-small cell lung carcinoma [61]. These results collectively indicate a pattern of aberrant activation of GLI1 in many forms of cancer, suggesting that GLI1 could be a potential target for treating these tumors. It is also necessary to analyze the roles of the different GLI1 mutations that are found in cancer on the development and progression of the cancer. There is also a potential for GLI1 to behave differently in tumors from different origins, or genetic backgrounds, and this needs to be assessed.

### 4.2. Role of Aberrant GLI1 in Pancreatic Cancer

Almost 90% of pancreatic ductal adenocarcinoma (PDA) has shown mutation in *KRAS* oncogene. Interestingly, KRAS is also one of the proposed non-canonical activators of GLI1. Furthermore, GLI1 regulates various proteins in PDA to induce carcinogenesis. One such protein is MUC5AC, a gel forming mucin, considered to be a biomarker for the poor prognosis in PDA patients [62]. It was found that MUC5AC is a direct transcriptional target of GLI1 and regulates its transcription by binding to the promoter through conserved CACCC-box-like cis-regulatory elements. Elevated MUC5AC, in turn, modulates β-catenin and E-cadherin which promote EMT. Another transcriptional target for GLI1 in pancreatic cancer was RegIV [63]. Increased expression of RegIV was observed in certain cancers and its expression is associated with increased proliferation, invasiveness and resistance to apoptosis. In the pancreatic tumor microenvironment, *KRAS* mutant cancer cells induced the expression of GLI1 in surrounding fibroblasts, followed by the binding of GLI1 to the cytokine interleukin (IL)-6 promoter inducing its expression [64]. In turn, IL-6 regulates signal transducer and activator of transcription 3 (STAT3), a transcription factor which is necessary for the development and progression of pancreatic cancer.

In contrast to the aforementioned studies, GLI1 was found to play a tumor protective role in mice crossed between *GLI1* knockout (GKO) and cre-dependent pancreatic activation of oncogenic *KRAS* with concomitant loss of the tumor suppressor *tp53* (KPC). Increased tumor growth was noticed in the GKO/KPC mice compared to KPC group alone. Further studies using ChIP analysis and functional assays revealed that the apoptosis inducing proteins, Fas and FasL, are the direct targets of GLI1 [65]. These results suggest that GLI1 induces apoptosis in response to oncogenic stress by regulating Fas and FasL to inhibit early carcinogenesis. However, downregulation of GLI1 did not attenuate the tumor growth at later stages of the tumor development in the same study. These observations open up the need for critical evaluation of GLI1 and its inhibition during various stages of the tumor development, which may change existing treatment modalities not only based on the tumor type but also based on the tumor progression.

### 4.3. Role of Aberrant GLI1 in Medulloblastoma 

Analysis of ChIP-sequencing data of GLI1 binding sites revealed different pattern of binding loci in granule neuron progenitors (GNP) compared to medulloblastoma [66]. The combined activation of GLI1 and atonal homolog 1 (ATOH1) (transcription factor) in primary GNP resulted in increased medulloblastoma formation. However, ATOH1 activation alone was not enough to induce medulloblastomas [67]. Additionally, GLI1 induces the expression of SNAIL1 transcription factor in GNP which in turn directly regulates the transcription of the oncogene *N-MYC*, which is hypothesized to mediate the oncogenic transformation of GNP into medulloblastoma [68]. Furthermore, GLI1 plays important role in the migration of cells derived from CD133-positive malignant glioma cells. Knocking down GLI1 in these cells significantly reduced their mobility [69]. It is important to highlight here again that in addition to GLI1 role in regulation of various mechanisms in tumor development, GLI1-induced disruption of ATR-CHK1 checkpoint signaling in the developing brain may generate the precursor lesions that lead to medulloblastoma formation.

### 4.4. Role of Aberrant GLI1 in Breast Cancer

Increased expression of GLI1 was also found in breast cancers compared to benign breast tissues [70]. In highly invasive triple negative breast cancer, the ErbB1/2-resistant cell line showed increased expression of GLI1 compared to the ErbB1/2-sensitive cell line. Downregulation of GLI1 in the ErbB1/2-resistant breast cancer cells reduced proliferation, increased apoptosis and decreased migration [71]. Microarray analysis of the invasive breast cancer tissue showed increased expression of GLI1 and forkhead box C2 (FOXC2), a transcription factor involved in embryonic development and tissue homeostasis. Patients with nuclear GLI1 or FOXC2 typically exhibited diminished length of survival [72]. Conditional induction of GLI1 expression in mouse mammary gland induced hyperplastic lesions, defective terminal end buds, and tumor development [73]. GLI1-induced tumors were largely heterogeneous in composition. These tumors expressed genes that are involved in proliferation and metastasis, as well as epithelial progenitor markers like keratin 6 and BMI1. Interestingly, silencing GLI1 expression in GLI1-induced tumors did not result in tumor regression, as the cells unexpectedly continued proliferation independent of GLI1. The promoter region of the HH gene was found to be hypo-methylated in breast cancers and this correlated with increased HH and nuclear factor (NF)-kB expression, and nuclear accumulation of GLI1 [74]. GLI1 also transcriptionally regulated the expression of the potent pro-angiogenic secreted molecule, cysteine-rich protein 61 (CYR61) in breast cancer, which resulted in highly angiogenic tumors that were spontaneously metastatic (Figure 3) [75].

### 4.5. Role of Aberrant GLI1 in Inducing EMT

EMT is considered to be an important feature in cancer development. This process allows the epithelial cells to undergo various biological changes transforming them to a mesenchymal cell phenotype characterized by enhanced migration, invasiveness and resistance to apoptosis. Cancer cells adapt this EMT transition to detach from the cell-cell adhesion, acquire invasive potential and undergo metastasis (Figure 3). SNAIL1 is one of the primary transcription factors that regulate the EMT transition. Expression of GLI1 induced the upregulation of SNAIL1 resulting in the EMT phenotype, including the loss of E-Cadherin in RK3E cells [76]. SNAIL1 was also found to interact with C-terminal binding protein 2 (CtBP2) a transcriptional co-repressor that promotes cancer cell migration and invasion (EMT) by inhibiting multiple tumor suppressor genes [77]. Furthermore, GLI1 binds to the promoter region of CtBP2 and increases its transcription.

Loss of E-cadherin causes β-catenin to migrate into the nucleus, where it acts as a transcription factor and induces cell transformation [78]. *In situ* hybridization analysis of colorectal samples showed increased expression of GLI1 mainly in the malignant crypts of adenocarcinomas. Analysis of normal, hyperplastic and malignant endometrium showed increased expression of GLI1, mostly in malignant endometrium. Similarly, β-catenin, an important member of the WNT signaling pathway, also showed increased expression in conjunction with GLI1 [79]. Ectopic expression of GLI1 induced β-catenin expression in the nuclei of endometrial cancer cell lines, and aberrant activation of this pathway may have a role in the development of endometrial cancer. Transplantation of GLI1 overexpressing pancreatic cancer cells into nude mice enhanced liver metastasis and intra splenic miniature metastasis. Microarray analysis of this system revealed that GLI1 regulates EMT promoting factors, including TGFβ, RAS, WNT, growth factors, phosphoinositide 3 kinase (PI3K)/AKT, integrins, transmembrane 4 superfamily (TM4SF), [80] and S100 calcium-binding protein A4 (S100A4) [81]. Ovarian cancer cells overexpressing GLI1 showed increased proliferation, mobility, invasiveness and up-regulation of E-cadherin and vimentin [82]. In contrast, increased expression of GLI1 in immortalized N/telomerase reverse transcriptase 1 (TERT1) keratinocytes induced stem cell characteristics like reduced EGFR, compact colony formation and repressed extracellular signal-regulated kinase (ERK) activity [83]. Overexpression of GLI1 failed to induce EMT in these cells and in fact, reduced the expression of vimentin, an EMT marker. This differential regulation of EMT by GLI1 might be due to the role of oncogenes. Unlike immortalized N/Tert1 keratinocytes, most of the normal immortalized cells which showed EMT phenotype upon GLI1 expression are immortalized with viral oncogenes.

### 4.6. Role of Aberrant GLI1 in Regulating hTERT

The reverse transcriptase hTERT is responsible for protecting the telomeres and for regulating replication of chromosomes in mammalian cells. Loss of hTERT induces cellular senescence, while increased hTERT has been observed in many cancer cells. The exogenous expression of GLI1 did not increase expression of hTERT in non-malignant cells; however, downregulation of GLI1 and GLI2 in human colon cancer, prostate cancer and glioblastoma multiforme by C-terminus truncated GLI3 repressor mutant (GLI3R), or by GANT61, reduced hTERT protein expression (Figure 3) [84]. Critical analysis of GLI1 and hTERT relationship in both normal and cancer cells of different lineages are needed to further establish GLI1’s role in carcinogeneis.

### 4.7. Role of Aberrant GLI1 in Epigenetic Regulation

Epigenetic regulation of various genes at specific times is controlled by DNA methyl transferases (DNMT). Aberrant expression of these DNMTs and dysregulation of DNA methylation has been reported in many cancers. Analysis of pancreatic cancer samples by immunohistochemistry has revealed an increased expression of GLI1, DNMT1 and DNMT3a in the cancer cells compared to normal cells [85]. Overexpression of GLI1 increased DNMT1 and DNMT3a levels, whereas GLI1 knockdown caused a decrease in their expression. ChIP analysis revealed that GLI1 binds to the *DNMT1* promoter suggesting that GLI1 may regulate tumor-related genes through DNMT1 expression. It will be interesting to determine whether regulation of various oncogenes by GLI1 is mediated by epigenetic regulation. Furthermore, validating the synergistic effect of both GLI1 and DNMT inhibitors in various tumor cells will give new treatment options.

### 4.8. Role of HH-Independent Aberrant GLI1 in Carcinogenesis

Studies in colorectal carcinomas revealed that GLI1 expression in these tumors were independent of HH expression [86]. Similarly, specific expression of GLI1 or GLI2 in the basal layer of murine epidermis induced BCC even in the presence of wild-type *PTCH1* alleles [87]. Recently, it has been shown that GLI1 expression in PDA is independent of the HH–PTCH–SMO signaling pathway and that it is essential for the proliferative and transformation potential of the tumor cells. Further analysis in this study revealed that GLI1 is regulated by TGFβ and KRAS. It has also been reported that GLI1 is over-expressed in more than 50% of hepatocellular carcinomas, and inhibition of HH signaling attenuated tumor growth and induced apoptosis [88]. Importantly, of all the HH signaling molecules analyzed in hepatocellular carcinoma, only GLI1 expression correlated with the poor prognosis in these patients [89]. These results confirm that GLI1 is the most significant member of the HH signaling pathway in promoting carcinogenesis. Furthermore, the mechanism by which oncogenes regulate GLI1 and how GLI1 affects oncogenes should be analyzed in detail. Specific cancer therapies to inhibit both the relevant oncogenes and GLI1 should be validated to design more effective treatment strategies.

### 4.9. Role of Aberrant GLI1 in Tumor Recurrence

Apart from the role of GLI1 in directly inducing carcinogenesis, it has been documented that GLI1 has a significant function in inducing tumor recurrence. In hepatocellular carcinoma patients, increased levels of PTCH1 and GLI1 mRNAs showed significant correlation with recurrence of the disease after surgical resection [90]. A similar pattern of tumor recurrence was also observed in GLI1 overexpressing glioma cells [91]. It will be interesting to note whether GLI1 alone is enough to induce tumor recurrence or if it works in combination with other oncogenic signals. Either way, examining GLI1 status will determine its potential as a biomarker for predicting post-resection disease recurrence.

### 4.10. Section Summary

GLI1 has an important role in the development and progression of multiple cancers. In light of its role in promoting carcinogenesis, it is vital to analyze the mechanisms through which GLI1 is regulated by oncogenes. Since the role of GLI1 varied within the different stages of tumor progression like hyperplasia, dysplasia, malignant and invasive tumor, the importance of oncogenes and their association with GLI1 at various stages of tumor progression has to be thoroughly analyzed. Dysregulation of cell cycle check points by aberrant GLI1 in normal cells is an important mechanism that has to be elucidated, because it may result in the accumulation of endogenous DNA damage which ultimately leads to GI. Critical analysis of the GLI1 regulation in different stages of the cell cycle will aid in developing appropriate preventive and treatment strategies. Furthermore, GLI1 has also been shown to have a potential role in tumor recurrence. Analysis of GLI1 status prior to treatment may allow development of tailored treatments to minimize incidence of tumor recurrence. GLI1 has diverse functions including regulation of epigenetic methylation, cancer stem cells, hypoxia and human telomerase.

## 5. Role of Aberrant GLI1 in Chemoresistance

In addition to promoting tumors, GLI1 also has a prominent role in inducing chemo-/radio-resistance of cancer cells through the regulation of multiple mechanisms. It is also evident from several studies that GLI1-expressing tumors are resistant to various chemotherapeutic agents; therefore, decoding the diverse mechanisms that GLI1 regulates to induce therapeutic resistance will aid in the development of more effective treatment strategies. It is important to note that GLI1 has a wide range of regulatory functions including modulating transport proteins, cell cycle checkpoints and modifying the anticancer drug activity to induce chemoresistance.

### 5.1. Role of Aberrant GLI1 in MDR

**Figure 4:**
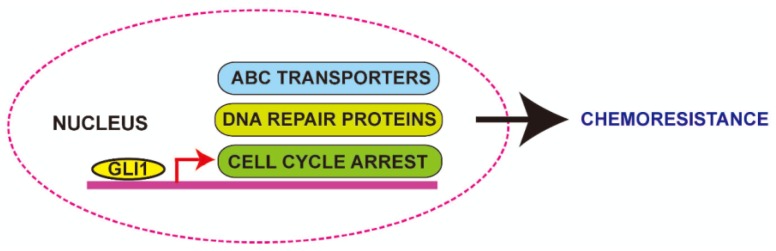
Role of glioma-associated oncogene homolog 1 (GLI1) in chemoresistance. GLI1 regulates the ATP-binding cassette (ABC) transporter family of proteins inducing drug efflux. Apart from this, GLI1 also induces the expression of proteins involved in cell cycle arrest and DNA damage repair. It is well known that these proteins repair the DNA damage induced by therapeutic agents while treating cancer cells and thus promotes cancer cell survival and facilitates chemoresistance.

In cluster of differentiation (CD)133-positive glioma cancer stem-like cells, which overexpress GLI1 [69], increased resistance to chemotherapeutic agents like temozolomide, carboplatin, paclitaxel and etoposide have been observed [92]. On the other hand, inhibition of GLI1 signaling enhances the sensitivity of CD133 cells to temozolomide [93]. Additionally, GLI1 overexpression-induced MDR has been correlated with the recurrence of glioma after chemotherapy [91]. Similarly, GLI1-induced MDR has been observed in chemoresistant pancreatic cancer cells and ovarian cancer cells [94,95]. Inhibition of GLI1 by GANT61 in pancreatic cancer cells has not only reduced its stemness but also sensitized the cells to genistein [96]. Mechanistically, it was shown that GLI1 induces drug resistance, at least partially through increasing drug efflux by ATP-binding cassette (ABC) transport related proteins like MDR1, ABCB1, P-glycoprotein, breast cancer resistance protein (BCRP) and ABCG2 (Figure 4) [97].

Similarly, GLI1 overexpression was found in the chemoresistant leukemia cancer cell line, Lucena-1. In these cells, increased expression of P-glycoprotein, a member of the ABC transporter family facilitates the efflux of harmful substances from the cells. Downregulation of GLI1 reduced the expression of P-glycoprotein and chemo-sensitized the cells [98]. Another member of the ABC transporter family (ABCG2) was found to be overexpressed in B-cell lymphoma, in which GLI1 is overexpressed (Figure 4). On the other hand, downregulation of GLI1 transcriptionally repressed ABCG2 and sensitized cancer cells to chemotherapeutic agents [99,100].

Cisplatin uptake in cells is primarily regulated by multiple transport proteins such as octamer-binding protein 1 (OCT1), OCT2 and OCT3 and also by the copper transporter CTR1, whereas efflux of cisplatin occurs through the ATPase copper transporter ATP7B. Inhibition of GLI1 in ovarian cancer cells that are resistant to cisplatin caused an accumulation of cisplatin in the nucleus [101]. Further analysis revealed that GLI1 binds to the promoter of all five of these genes responsible for cisplatin transport, thus playing a major role in regulating these proteins and tumor resistance to platinum drugs. Overall, most of the GLI1-induced chemoresistance is mediated by the direct regulation of MDR genes. Inhibition of GLI1 not only suppresses tumor growth, but also sensitizes the cancer cells to chemotherapeutic agents. Hence combination of GLI1 inhibitors with established chemotherapeutic agents could be an option for clinical testing.

Reflux-induced injury is one of the risk factors that cause esophageal carcinoma. Ursodeoxycholic acid and aspirin are used to suppress the reflux-induced injury. GLI1 is overexpressed in esophageal carcinoma where it downregulates the cell cycle related protein CDK2. Interestingly, it was found that the Urso-Aspirin combination downregulates GLI1 expression in esophageal cancer there by restoring CDK2 levels [102]. Supplementing the tumor cells with cholecalciferol, a precursor of active vitamin D, blocked the GLI1 activation, inducing cell death and tumor regression in these cells [103].

### 5.2. Role of Aberrant GLI1 in Modifying the Drug Activity

Recently, it has been shown that in acute myeloid leukemia (AML) patients, GLI1 expression induces resistance to ribavirin by modifying the activity of the drug [104]. GLI1 alters ribavirin by promoting the addition of glucuronic acid through a process called glucuronidation mediated by the drug-modifying enzyme UDP-glucuronysltransferase1A (UGT1A). The addition of the glucuronic acid moiety enhances the hydrophilicity of the drug and promotes its efflux. Knocking down GLI1 affects the protein stability of UGT1A and sensitizes the cells to ribavirin. It will be interesting to determine if other commonly used chemotherapeutic agents are modified by GLI1, and if so, whether it is limited to glucuronidation or if other mechanisms are used.

### 5.3. Role of Aberrant GLI1 in Radio-Resistance

Apart from chemotherapy, GLI1 overexpression also induces cellular resistance to IR [105]. In a recent study, hypoxic renal cell carcinoma cells showed increased expression of HH-independent GLI1, which is mediated by hypoxia-inducible factor 2a (HIF2a). Irradiation of GANT61-treated hypoxic cancer cells showed less DNA repair compared to control hypoxic cells and sensitized them to radiation. These findings show that DNA repair is not compromised in the GLI1 overexpressed cells that are irradiated and thus becomes resistant to radiation. Downregulation of GLI1, or HIF2a, or both sensitizes cancer cells to radiation. Thus by regulating various signaling molecules and transport proteins, as well as by modifying the chemotherapeutic drugs themselves, GLI1 induces chemoresistance in a variety of tumors.

### 5.4. Inhibitors of Aberrant GLI1

Due to the role of HH signaling in carcinogenesis, tumor progression and chemoresistance, inhibitors targeting different members of the pathway have been developed. One example is Robotnikinin a small molecule developed to target HH ligand for the treatment of cancer [106]. Inhibition of SMO prevents UV-induced basal cell carcinomas through the regulation of Fas expression and apoptosis [107]. Several small molecule inhibitors have been developed that target SMO. Cyclopamine and its synthetic analogue saridegib are examples of the SMO inhibitors that are currently in clinical trials [108]. Vismodegib was the first SMO inhibitor to be approved by the FDA for the treatment of BCC [109]. Vismodegib is also in clinical trials for the treatment of colorectal cancer, small cell lung cancer and several other cancers that exhibit activated HH signaling pathway. However, there are also studies that failed to demonstrate the effectiveness of SMO inhibitors. For example, cyclopamine was not able to complement the GLI1 activated by osteopontin through a non-canonical pathway [110]. Taken together, these findings suggest that even though many inhibitors are available to target the upstream members of GLI1 in HH signaling, the agents that selectively inhibit transcriptional activity of GLI1 (the final step in the signaling pathway) may be the most effective inhibitors of HH signaling in cancer cells. Currently, several small molecule inhibitors which are known to suppress the transcription activity of GLI1, such as HPI (1–4), GANT58, GANT61 and arsenic trioxide are being developed.

### 5.5. Section Summary

As we discussed earlier, targeting GLI1 (final step) may be the better approach to inhibit HH signaling. While the GLI1-specific inhibitors are still in preclinical development stages, there are studies showing that GLI1-expressing tumors are resistant to numerous chemotherapeutic agents. The best approach to solve this problem may be to use combinational drug therapy utilizing both GLI1 inhibitors and established chemotherapeutic agents. Synergistic effects were observed when RMS tumors were treated with the combination of GLI1 inhibitors and chemotherapeutic agents, such as temsirolimus or vincristine [111,112]. Pathway-specific inhibitors like AKT inhibitors [113], PI3K inhibitor [114] can also be used to target cancer cells in conjunction with GLI1 inhibitors.

## 6. Conclusions and Future Perspectives

Overall, our review summarizes and updates the role of aberrant GLI1 activation in the DNA damage response, carcinogenesis and chemoresistance. While the primary function of GLI1 is in embryonic development, it is aberrantly activated in many cancers. The controlled activation and repression of GLI1 during embryonic development has to be analyzed in detail as this may shed light on new mechanisms to regulate this protein. The results may provide new directions for targeting aberrant GLI1 expression in cancer treatment. Another area that needs to be examined is the regulation of GLI1 by oncogenes in a non-canonical pathway. Careful elucidation of the HH-independent pathways through which oncogenes activate GLI1 and how GLI1 impacts these oncogenes is required in order to develop effective treatment strategies for GLI1-dysregulated tumors. In a seminal article, Hanahan and Weinberg proposed six hallmarks of cancer in the year 2000 [115]. In this review we have attempted to document the role of GLI1 in those six hallmarks such as: sustaining proliferative signaling, evasion of growth suppressors, resistance to cell death, enabling of replicative immortality, induction of angiogenesis, and activation invasion and metastasis. In 2011, the authors appended two new hallmarks of cancer, dysregulated cellular metabolism and avoiding immune destruction [116]. Even though GLI1 is indirectly implicated in regulating the altered metabolism and immune evasion of cancer cells, direct association has not been documented yet. Additional reports in the future implicating GLI1 in the direct regulation of the updated hallmarks of cancer are very possible. Apart from these hallmarks, other important functions like epigenetic modification, angiogenesis, hypoxia, cancer stem cells, hTERT activity, DNA damage, repair and GI are induced or regulated by GLI1. Another important and provocative function of aberrant GLI1 is the regulation of cell cycle checkpoints. Activation of cell cycle checkpoints in cancer cells by GLI1 induces chemoresistance. Whereas, dysregulation of cell cycle checkpoints in normal cells by aberrant GLI1 may results in GI. Analysis of the differential regulation of cell cycle checkpoints by GLI1 in normal and cancer cells has to be studied in detail. Additionally, analyzing the roles of tGLI1 in tumor recurrence, cell cycle regulation, chemoresistance and DDR will aid in understanding the role of GLI1 in cancer. Even though HH inhibitors show successful anti-tumor activity in BCC and medulloblastoma, unfortunately, the clinical trials for the HH inhibitor vismodegib showed no effect in progressive pancreatic cancer compared to the placebo [117]. As GLI1 is known to be regulated by other oncogenic signals independently of HH signaling, the role of these oncogenic signals in pancreatic cancer and its regulation of GLI1 in various stages of cancer should be analyzed in detail to overcome the limitations. Inhibiting GLI1 alone or in combination with oncogenic signals will help to solve the complex roles of GLI1 in carcinogenesis. Further, we believe that developing novel GLI1-specific inhibitors and finding the correct synergistic agents to kill the cancer cells would be the ideal approach for future study. However, the potential toxicity or side effects of the combinatorial treatment should be validated prior treatment.
